# Efficacy and safety of transcranial pulse stimulation in young adolescents with attention-deficit/hyperactivity disorder: a pilot, randomized, double-blind, sham-controlled trial

**DOI:** 10.3389/fneur.2024.1364270

**Published:** 2024-05-09

**Authors:** Teris Cheung, Benjamin K. Yee, Bolton Chau, Joyce Yuen Ting Lam, Kwan Hin Fong, Herman Lo, Tim Man Ho Li, Albert Martin Li, Lei Sun, Roland Beisteiner, Calvin Pak Wing Cheng

**Affiliations:** ^1^School of Nursing, The Hong Kong Polytechnic University, Kowloon, Hong Kong SAR, China; ^2^The Mental Health Research Centre, The Hong Kong Polytechnic University, Kowloon, Hong Kong SAR, China; ^3^Department of Rehabilitation Sciences, The Hong Kong Polytechnic University, Kowloon, Hong Kong SAR, China; ^4^Department of Applied Social Sciences, The Hong Kong Polytechnic University, Kowloon, Hong Kong SAR, China; ^5^Department of Psychiatry, The Chinese University of Hong Kong, Shatin, Hong Kong SAR, China; ^6^Department of Paediatrics, The Chinese University of Hong Kong, Shatin, Hong Kong SAR, China; ^7^Department of Biomedical Engineering, The Hong Kong Polytechnic University, Kowloon, Hong Kong SAR, China; ^8^Department of Neurology, Vienna Medical University, Vienna, Austria; ^9^Department of Psychiatry, The University of Hong Kong, Pokfulam, Hong Kong SAR, China

**Keywords:** efficacy, transcranial pulse stimulation, rct, ADHD, neuromodulation, adolescents

## Abstract

**Background:**

This is the first study to evaluate the efficacy and safety of transcranial pulse stimulation (TPS) for the treatment of attention-deficit/hyperactivity disorder (ADHD) among young adolescents in Hong Kong.

**Methods:**

This double-blind, randomized, sham-controlled trial included a TPS group and a sham TPS group, encompassing a total of 30 subjects aged 12–17 years who were diagnosed with ADHD. Baseline measurements SNAP-IV, ADHD RS-IV, CGI and executive functions (Stroop tests, Digit Span) and post-TPS evaluation were collected. Both groups were assessed at baseline, immediately after intervention, and at 1-month and 3-month follow-ups. Repeated-measures ANOVAs were used to analyze data.

**Results:**

The TPS group exhibited a 30% reduction in the mean SNAP-IV score at postintervention that was maintained at 1- and 3-month follow-ups.

**Conclusion:**

TPS is an effective and safe adjunct treatment for the clinical management of ADHD.

**Clinical trial registration:**

ClinicalTrials.Gov, identifier NCT05422274.

## Introduction

Local epidemiological data in Hong Kong suggest that attention-deficit/hyperactivity disorder (ADHD) affects approximately 6% of children and that it is twice as common in males than in females (1). The prevalence of ADHD in adults is approximately 2.5% (2). ADHD is characterized by persistent symptoms of inattention and/or hyperactivity/impulsivity (3) that emerge in childhood (4). These symptoms may persist into adulthood, leading to poor life outcomes and affecting employment and interpersonal relationships (5). ADHD may affect all aspects of an individual’s life and has a negative impact on family members (6). The neurobiological mechanism of ADHD may be attributed to dopaminergic imbalance in the forebrain and basal ganglia. The prefrontal cortex, anterior cingulate cortex, insula, amygdala and cerebellum are also linked to ADHD pathophysiology (7). Typical ADHD treatments include pharmacotherapy, with stimulant medications (e.g., methylphenidate, amphetamine) and nonstimulant medications (e.g., atomoxetine) targeting dopaminergic and noradrenergic systems in the frontal cortex and the dopaminergic system in the basal ganglia (8). These drugs are effective and safe for the majority of patients; however, 20% of patients do not tolerate these medications or fail to respond (9). Although these medications can significantly improve ADHD symptoms and patient outcomes, long-term drug compliance is necessary to sustain treatment efficacy (10). Medication dosages also need to be individually monitored to minimize adverse effects while maintaining efficacy (8). It remains debatable whether the long-term benefits of taking medications outweigh the risks in individuals with ADHD.

Medication (e.g., methylphenidate) is usually the first line of pharmaceutical treatment for ADHD symptoms among adolescents, however; medication adherence and its long-term efficacy is always questionable, partially attributed to medication non-adherence and drug attitude. This claim was supported by the results reported by a cross-sectional study (11) comprising 181 adolescents aged 12–18 years old. Half of the study population (*n* = 93; 51%) experienced side effects, such as decreased appetite and sleep problems. Most participants (*n* = 150; 83%) had an indifferent attitude which referred to perceived low necessity and low concerns toward their ADHD medication. More than half of the study population (*n* = 111; 61%) reported ‘nonadherent’ toward their prescribed medications and thus, researchers work ameliorate hard to investigate other non-pharmaceutical options for this clientele.

Although mindfulness-based cognitive therapy (MBCT) has recently been demonstrated to be an effective psychosocial intervention (12), the long-term sustainability of the benefits of these psychosocial interventions on ADHD has yet to be confirmed. In fact, pharmacotherapy is not considered a monotherapy for more than 50% of adult ADHD cases (13, 14), and a combination of cognitive behavioral therapy (CBT) and medication yields broader improvements in executive functioning than CBT alone.

It is evident that exiting NIBS *s*tudies have used EEG-neurofeedback, trigeminal nerve stimulation (TNS), rTMS, and tDCS in different age groups but have reported inconsistent results in individuals with ADHD. Almost all NIBS studies focused on the left/right/bilateral DLPFC in individuals with ADHD. Stimulation targeting the right inferior frontal cortex (rIFC) was shown to be ineffective. Since ADHD is increasingly prevalent in Hong Kong, there is a pressing need to evaluate the efficacy of the latest NIBS technology (i.e., transcranial pulse stimulation, TPS) which has not been tested nationwide. Findings emerge will provide new neuroscientific evidence to determine whether TPS is an effective adjunct treatment for ADHD in clinical psychiatry. Neurobiological mechanism of ADHD may be attributed to dopaminergic imbalance in the forebrain and basal ganglia. The prefrontal cortex, anterior cingulate, insula, amygdala and cerebellum are also linked to ADHD pathophysiology. As plastic cortical changes are considered to be the substrate of learning and memory, both in development and aging, an overview of the relevant literature about neuroplasticity and its modulation in physiological and pathological conditions is mandatory also in adults [for example see (15, 16)], nonetheless, the scope of this paper is to focus on the efficacy of TPS on young adolescents aged between 12 and 17 years old, in particular, we focus on participants’ behavioral and cognitive changes after TPS interventions using participants’ self-reported data. Although we also aimed to investigate the different neural substrates underpinning neuropsychological performance in our participants in terms of their attention performance, executive memory, and intra-individual variability (IIV) in reaction time. Nonetheless, the intercept of neurophysiological substrates of ADHD with TPS will only be discussed in a separate paper using MRI data analysis [Cheung et al. (17), under review].

### Neuromodulation and non-invasive brain stimulation (NIBS)

Designing interventions that could directly modulate brain function has received increasing interest with the development of technology capable of delivering narrow and tailored modulation of specific brain circuits. Noninvasive brain stimulation (NIBS), such as repeated transcranial magnetic stimulation (rTMS) and transcranial direct current stimulation (tDCS), is widely applied with the aim of rebalancing neural activity at the circuit level to normalize functions and behavior. Currently, these NIBS techniques are being used diagnostically and therapeutically for different types of neurodegenerative diseases (e.g., Alzheimer’s disease, Parkinson’s Disease) (18), pediatric epilepsy (19), neuropsychiatric disorders (e.g., attention-deficit/hyperactivity disorder, major depressive disorder, substance use disorder) (20) and neurodevelopmental disorders (e.g., autism) (21). A recent systematic review and meta-analysis (22) of neurotherapeutics for ADHD provided evidence that electroencephalography (EEG)-neurofeedback showed small/medium effects compared to nonactive controls in randomized controlled trials. However, trials evaluating rTMS or tDCS have reported mixed outcomes. Findings regarding rTMS-induced improvements in cognition or symptoms in individuals with ADHD have been inconsistent, while tDCS studies targeting the dorsolateral prefrontal cortex (DLPFC) led to small cognitive improvements in individuals with ADHD. The key findings in specific age groups (e.g., children, adolescents, and adults) of people with ADHD are summarized below (see Table 1).

**Table 1:** Findings of non-invasive brain stimulation (NIBS) studies on ADHD.

Authors	N	Age	Design	Session/duration	Treatment region	Results
*Transcranial direct current stimulation (tDCS)*						
Cosmo et al. (19)	60	18–65	Double-blind, sham-controlled RCT	1	Left DLPFC	No significant differences in ADHD symptoms between the tDCS & sham group
Soff et al. (20)	15	12–16	Double-blind RCT	5	Left DLPFC	Significant reduction of Hyperactivity & Inattention (*p* < 0.05) but no effect on impulsivity
Allenby et al. (4)	37	18–65	Double-blind, sham-controlled RCT	3	Left DLPFC	tDCS improved impulsivity symptoms
Leffa et al. (21)	64	18–60	Double-blind, parallel, sham-controlled RCT	20	Anodal-right and cathodal-left prefrontal	Mean inattention score was 18.88 (SD 5.79) in the active tDCS group compared with 23.63 (SD 3.97) in the sham tDCS. Significant treatment by time intervention evaluated by clinician-administered version of the adult ADHD self-report scale (*β* interaction: −3.18, *p* < 0.001).
Westwood et al. (22)	50	10–18	Double-blind, sham-controlled RCT	15	rIFC	No significant improvement in core ADHD symptoms (*p* > 0.05)
*Repetitive transcranial magnetic stimulation (rTMS)*						
Paz et al. (23)	22	12–16	Single-blind RCT	20	Bilateral DLPFC	No effect on clinical/cognitive outcomes (*p* > 0.05)
Cao et al. (24)	64	6–13	3-armed RCT rTMS (*n* = 20); ATX (*n* = 19); rTMS+ATX (*n* = 21) *ATX = Atomoxetine	6 weeks	Right DLPFC	rTMS+ATX group improved significantly in inattention & hyperactivity/impulsiveness at posttreatment (*p* < 0.05). All groups showed improvements in clinical/cognitive measures.
*Trigeminal nerve stimulation (TNS)*						
McGough et al. (25)	62	8–12	Double-blind, sham-controlled RCT	4 weeks	Right frontal lobe and frontal midline	Significant reduction of ADHD-RS score (*p* = 0.005) and CGI score on active TNS group (*p* = 0.003) compared to sham TNS group

DLPFC, dorsal lateral prefrontal cortex; IFC, inferior frontal gyrus; RCT, randomized controlled trial.

In summary, previous NIBS studies have used EEG-neurofeedback, trigeminal nerve stimulation (TNS), rTMS, and tDCS in different age groups but have reported inconsistent results in individuals with ADHD. Almost all NIBS studies focused on the left/right/bilateral DLPFC in individuals with ADHD. Stimulation targeting the right inferior frontal cortex (rIFC) was shown to be ineffective (23). Since ADHD is increasingly prevalent in Hong Kong, there is a pressing need to evaluate the efficacy of the latest NIBS technology **(i.e., transcranial pulse stimulation, TPS)**. Such research would not only generate new neuroscientific evidence but also reveal whether TPS is an effective adjunct treatment for ADHD. If so, TPS treatment could reduce the global disease burden and psychiatric morbidities (e.g., mood disorders/anxiety disorders, eating disorders, and substance-related disorders) (24, 25) in Hong Kong.

### Mechanisms of TPS

TPS uses repeated single ultrashort pulses in the ultrasound frequency range to stimulate the brain. With a neuronavigation device, TPS can target specific and precise areas of the human brain (27). TPS differs from tDCS and rTMS because it does not involve direct or induced electric current. Using electric currents to stimulate the brain may be limited by conductivity (28) and failure to reach deep brain regions (29). In contrast, TPS uses low-intensity focused ultrasound, which provides good spatial precision and resolution to noninvasively modulate subcortical areas, addressing the problem of skull attenuation (30). By using lower ultrasound frequencies, TPS can stimulate deep cerebral regions, reaching as far as 8 cm into the brain. In other words, TPS can improve skull penetration in the human brain and improve treatment outcomes (27). Our theoretical basis is based on the biological mechanism of TPS. Mechanotransduction is the basic mechanism of transcranial pulse stimulation. Mechanotransduction is a biological pathway through which the cells convert the mechanical TPS stimulus into biochemical responses, thereby triggering some fundamental cell functions, such as migration, proliferation, differentiation and apoptosis (31). TPS can promote new blood vessel formation (angiogenesis) and nerve regeneration, stimulate vascular growth factors (32, 33) and brain-derived neurotrophic factor (34) and improve cerebral blood flow. TPS can stimulate deep cerebral regions (i.e., 8 cm) into the brain. The ultrashort ultrasound pulse could enhance cell proliferation and differentiation in cultured neural stem cells, which plays an important role in brain function repair in central nervous system diseases (35). TPS may affect neurons and induce neuroplastic effects, which increase cell permeability (35) stimulate mechanosensitive ion channels and release nitric oxide that causes vasodilation, increased metabolic activity and angiogenesis (36). TPS may play an important role in the restoration of brain function in individuals with CNS diseases (35).

### Previous research on transcranial pulse stimulation (TPS)

Application of ultrasound to the brain is a revolutionary therapeutic approach for patients with neuropsychiatric symptoms (37, 38). Since transcranial pulse stimulation (TPS) is a relatively new noninvasive brain stimulation (NIBS) technology, only four studies thus far have been conducted in clinical populations. The first study included 35 Austrian older adults with Alzheimer’s disease (AD) who were treated with three TPS sessions per week (6,000 pulses each; global brain stimulation) for 2–4 weeks. Participants showed significant improvement in the Consortium to Establish a Registry for Alzheimer’s Disease (CERAD) neuropsychological battery scores immediately after the intervention and at 1 month and 3 months after the intervention. The functional magnetic resonance imaging (fMRI) results also showed significantly increased connectivity within the memory network (27). Participants’ depressive symptoms were also significantly improved, as measured by the Geriatric Depression Scale (GDS) (*p* = 0.005) and Beck Depression Inventory (BDI) (*p* < 0.0001) at the 1-month and 3-month follow-ups compared with the baseline scores (27). The second TPS study was an open-label single-blind pilot RCT using waitlist control (WC) (39). This study evaluated the efficacy of TPS in people with MDD. A total of 30 subjects (aged 18–51 years) received 6 TPS sessions (400 TPS pulse/session) administered over 2 weeks on alternate days (total TPS pulse: 2,400; frequency: 2.5–3.0 Hz). Significant improvements in depression severity were observed in the TPS group compared with the WC group (*p* = 0.02), and the effect size was very large (Cohen’s *d* = −0.9) (39). However, these two studies were uncontrolled studies or open-label RCTs without a sham control group. Placebo effects must be considered when interpreting the results. The third study was a double-blind, randomized, sham-controlled trial evaluating the efficacy of TPS for autism spectrum disorder in 32 young adolescents (27 males) aged between 12 and 17 years in Hong Kong (40, 41). This trial used the same stimulation protocol (energy level: 0.2–0.25 mJ/mm2, pulse frequency: 2.5–4.0 Hz, 800 pulses/session) over 2 weeks on alternate days, but the ASD trial targeted the rTPJ. TPS over the right temporoparietal junction (rTPJ) was effective in reducing the core symptoms of ASD, as evidenced by a 24% reduction in the primary outcome, the Childhood Autism Rating Scale (CARS) score, in the TPS group. Additionally, there was a 53.7% reduction in the CGI total score in the TPS group at the 3-month follow-up compared with baseline values.

To date, there have been no further attempts to apply TPS to treat other neurodevelopmental disorders in children or young adolescents in Hong Kong or China. The impetus of our research was to fill this research gap, providing findings that could be crucial for ADHD symptom management.

### Objectives and hypotheses

The aims of this study were as follows: (i) to evaluate the efficacy and safety of TPS in young adolescents (aged 12–17 years) with ADHD in Hong Kong; (ii) to examine the associations of TPS with ADHD core symptom severity, executive function, inattention, hyperactivity, impulsivity, and oppositional defiance; and (iii) to examine brain functional connectivity changes after 2 weeks of TPS treatment by neuroimaging data. Based on our recent TPS study on ASD young adolescents, we hypothesized the expected outcomes as follows:

### Primary hypothesis

The TPS group will have a 30% reduction in the Swanson, Nolan, and Pelham Teacher and Parent Rating Scale (SNAP-IV) score (i.e., inattention, hyperactivity/impulsivity and oppositional defiance) after 2 weeks of TPS treatment compared with the sham TPS group, and this reduction will be maintained at the 1-month and 3-month follow-ups. We set up the hypothesis of 30% improvement of ADHD symptoms in the TPS group is based on a similar published double-blinded RCT using TPS on Autism Spectrum Disorder (ASD).

### Secondary hypotheses

1. Young adolescents with ADHD in the TPS group or the sham TPS group will have <5% increase in somatic discomfort in the 2-week TPS intervention.
2. The TPS group will have 30% improvement in ADHD symptoms and behavior compared with the sham TPS group after 2 weeks of TPS treatment, and this improvement will be maintained at the 1-month and 3-month follow-ups.
3. The TPS group will have 30% improvement in executive function after 2 weeks of TPS treatment compared with the sham TPS group, and this improvement will be maintained at the 1-month and 3-month follow-ups.
4. The TPS group will have 30% improvements in attention deficit, hyperactivity and impulsivity after 2 weeks of TPS treatment compared with the sham TPS group, and this improvement will be maintained at the 1-month and 3-month follow-ups.
5. The TPS group will have more brain connectivity changes after 2 weeks of TPS compared with the sham TPS group, and this difference will be maintained at the 1-month and 3-month follow-ups.

We set up the hypothesis of 30% improvement of ADHD symptoms in the TPS group was based on a similar published double blinded RCT using TPS on young adolescents (age 12–18) with Autism Spectrum Disorder (41) which led to a 24% reduction in the Childhood Autism Rating Scale (CARS), the primary outcome of this trial (41). We speculated the 5% increase in discomfort for the sham TPS group was based on the following arguments:

1. headache/pain is the most common adverse effect reported in three trials: (1) TPS randomized, sham-controlled trial on young adolescents with Autism Spectrum Disorder (41); (2) TPS randomized controlled trial using waitlist control on patients with Major Depressive Disorder (age 18–65) (39); and (3) the first open-label study that tested the efficacy of TPS on older adults (age 65+) with Alzheimer’s Disease (27). These three published trials had a < 4% adverse effect in either the TPS group or the sham group/waitlist control group.
2. There is cumulative evidence suggesting that placebo effect is a neurobiological phenomenon in different methodological approaches (42). In this study, participants in the sham TPS group may have the belief/desire that they were being administered a verum TPS and such expectation of this treatment may create uncertainty about the sensory information of pain/discomfort, leading to a placebo effect of a perceptual error (43, 44).

### How was the protocol determined in this study?

The first TPS study nationwide was conducted on 35 adult patients with Alzheimer’s disease and researchers used 6,000 TPS ultrashort ultrasound pulse (energy level: 0.2–0.3 mJmm −2; pulse frequencies of 1–5 Hz pulse per second) on each patient in each session throughout the 2-weeks’ interventions (27). Only 4% reported adverse effects during TPS but none required pain analgesics or other treatment.

Prior to this study, we also adopted a similar double-blind, randomized, sham-controlled trial on young adolescents with autism spectrum disorder (aged 12–17 years), but we used 800 TPS pulse (energy level: 0.2–0.3 mJmm −2; pulse frequencies of 2–4 Hz pulse per second) in each session, administered in 6 sessions spanning across 2-week period, as we only targeted on the rTPJ (right temporoparietal junction). Only 1/3 of participants out of 15 in the TPS group reported transient headache on a numerical pain score of 3–5 out of 10 but none of these participants required any pain analgesics after the intervention (41).

In this study, we also targeted on young adolescents aged between 12 to 17 years old with ADHD, but this time we targeted on the left DLPFC as the treatment region, the project team decided to adopt the same protocol as used in our ASD study. Both ASD and ADHD study had sought safety approval with the TPS expert team including the TPS manufacturer, neurologist, and mathematician within the Project team (27).

## Methods

### Trial design

**Figure 1:**
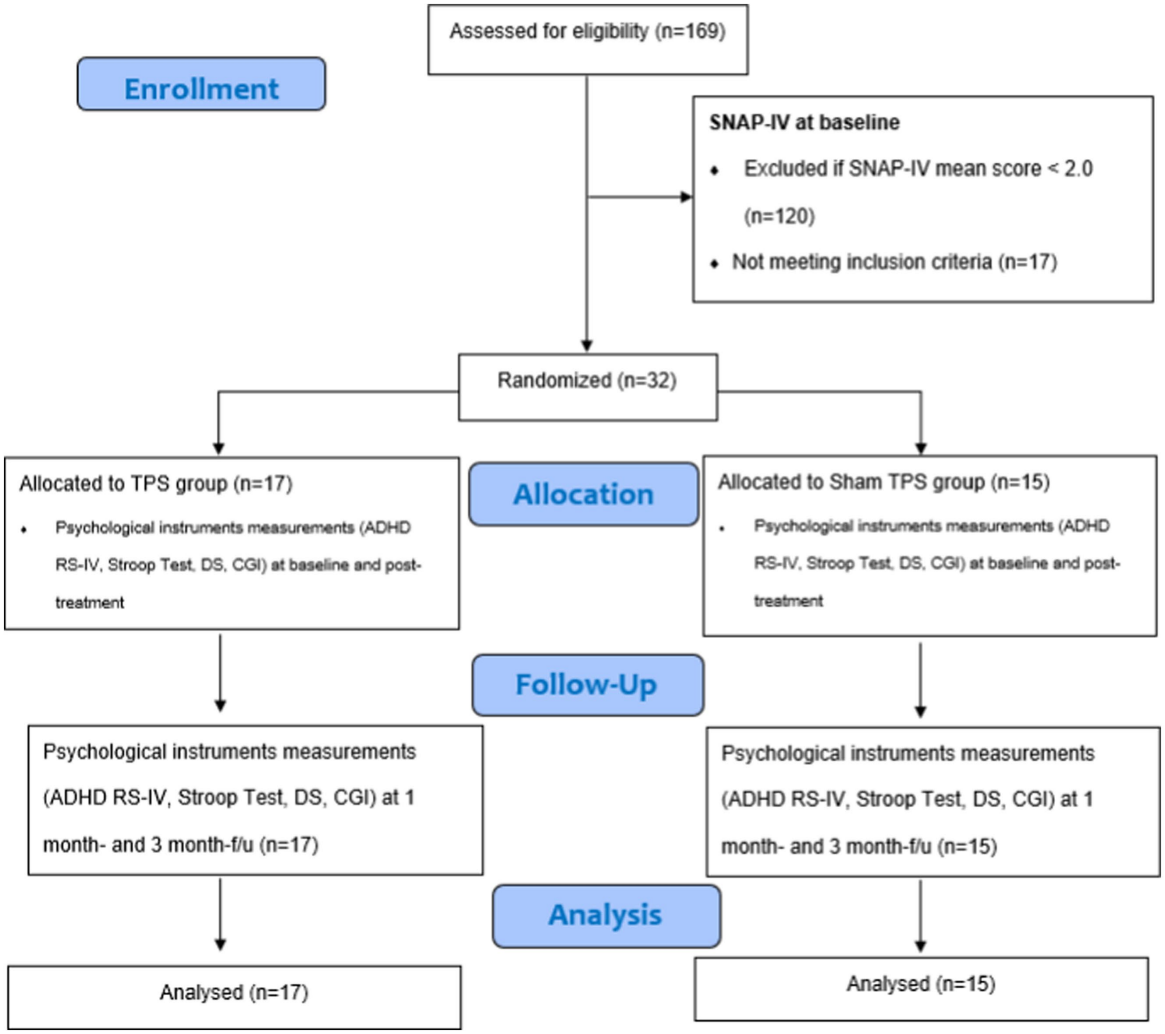
CONSORT diagram of procedures. SNAP-IV, Swanson, Nolan, and Pelham Teacher and Parent Rating Scale; ADHD RS-IV, ADHD Rating Scale–IV; DS, Digit Span; CGI, clinical global impression.

This study was a two-armed, randomized, double-blind, sham-controlled trial that evaluated the efficacy and safety of 2 weeks of TPS for treating ADHD among young adolescents. The trial design complied with the Consolidated Standards of Reporting Trials (CONSORT) statement (45). Participants were randomly allocated to the TPS group or sham TPS group. All parents of participants were informed about the randomization procedures and that their children had a 50% chance of receiving the TPS or the sham TPS treatment. This study was conducted in accordance with the Declaration of Helsinki (46). Both groups were assessed at baseline (T1), immediately after the 2-week intervention (T2), and at the 1-month and 3-month follow-ups (T3, T4) (47) (Figure 1).

### Subjects

Participants were recruited via a mass email invitation attached to a poster with a QR code that was delivered by collaborators in the Hong Kong Association for ADHD, CUHK, and HKU. A poster with a QR code was also posted in communal areas on campus. The recruitment period was 1 June to 30 September 2022.

### Inclusion and exclusion criteria

The inclusion criteria were as follows: (i) SNAP-IV score ≥ 2; (ii) confirmed diagnosis of ADHD according to the Diagnostic and Statistical Manual of Mental Disorders, 5th edition (DSM-5); (iii) Han Chinese ethnicity, aged 12–17 years, with no other mental disorders (e.g., intellectual disability disorder) or organic brain diseases that affect cognitive functions; (iv) no severe systemic diseases including heart, liver, lung, and kidney diseases; (v) IQ >80 according to the Stanford-Binet Intelligence Scales, 5th edition (SB-5); and (vi) written parental consent for TPS treatment and neuroimaging.

The exclusion criteria were as follows: (i) did not take ADHD medications in the past 2–4 weeks; (ii) received TMS/rTMS/tDCS or electroconvulsive therapy in the past 12 months; (iii) use of monoamine oxidase inhibitors in the past 14 days; (iv) a history of epilepsy, brain trauma, brain surgery/brain tumor, brain aneurysm or other concomitant unstable major medical conditions such as haemophilia or other blood clotting disorders or thrombosis; (v) communicative impairment; (vi) metal implants in the brain treatment region/artificial cardiac pacemaker; (vii) use of corticosteroids within the last 6 weeks before the first TPS treatment; or (viii) history of micro-cavernomas.

### Sample size

To our knowledge, no prior interventional study has evaluated the efficacy of TPS for ADHD. Based on our previous open-label pilot RCT (39) evaluating the use of TPS in adults with MDD that showed a large effect size (*d* = 0.91), we predicted that we would observe a large effect of TPS in this study. We used G*Power version 3.1.9.4 to calculate the target sample size. With a statistical power of 95%, a significance threshold of 0.05, a medium between-group effect size (*d*) of 0.91, and 4 measurement time points, we calculated that each group would need to include 15 subjects. Thus, a total sample size of 30 was needed. The attrition rate in our pilot MDD trial was 0%. We expected that the attrition rate in this ADHD trial would be <5%. Subjects who dropped out of the 2-week intervention period were replaced by another enrolled subject in this pilot study.

### Screening and self-administered questionnaire

The parents of participants completed an online application (accessed via QR code) that collected information on sociodemographic characteristics (age, sex, educational background, monthly family income, living circumstances, school year, participant’s psychiatric history and duration of ADHD diagnosis (in years/months), age at diagnosis, duration of prescribed medication use (in years/months), current drugs and dosages, and family history of psychiatric disorders).

Eligible subjects then completed the screening tool (the Swanson, Nolan, and Pelham Teacher and Parent Rating Scale; SNAP-IV). Those with a mean SNAP-IV score ≥ 2 were included. Subjects’ medical history, treatment regimen, and developmental history were obtained by direct enquiry with subjects’ parents by online interview prior to neuroimaging and TPS treatment. Both participants and parents were interviewed by the PI and the research personnel. Parents were asked to provide a valid medical certificate of their children’s ADHD diagnosis and prescribed formulation sheet during the online interview. Any parents who failed to provide these documents were not invited to participate in this trial.

### Randomization, allocation, and masking

All consenting participants were listed in alphabetical order according to their surnames, and each participant was assigned a unique identifier. Participants and their parents were informed that this study involved random allocation to a sham or treatment group. An independent statistician used a computer-generated list of random numbers (www.random.org) to ensure concealment of randomization. Randomization was conducted by an independent statistician off-site using a stochastic minimization programme to balance the sex, age and SNAP-IV scores of the participants. Block randomization with blocks of 10 participants (total: 3 blocks) was used to allocate treatment groups. Participants from each block were randomly assigned to the TPS group or the sham TPS group at a 1:1 ratio. To avoid information flow, participants/parents and research associates were blinded to group allocation to minimize potential contamination of the effects of TPS or subject bias. The experimenter was not involved in data collection or pre- and post-TPS measurements. Outcome measurements were collected by a research associate not involved in group allocation. Participants and their parents were asked to guess their group (TPS vs. sham TPS) in the last TPS session to determine the probability of guessing the group allocation correctly and thereby assess subject blinding (48).

### Intervention

TPS intervention was performed at the Integrative Health Clinic at the Hong Kong Polytechnic University (PolyU). A licensed mental health practitioner delivered the intervention. In this trial, we targeted the left dorsolateral prefrontal cortex (DLPFC). This brain region was selected based on previous tDCS research showing that the left and right DLPFC (49) are primarily the brain treatment regions for ADHD and that stimulation of the left DLPFC, specifically, can improve inattention and hyperactivity (4, 26).

### TPS procedures

**Figure 2:**
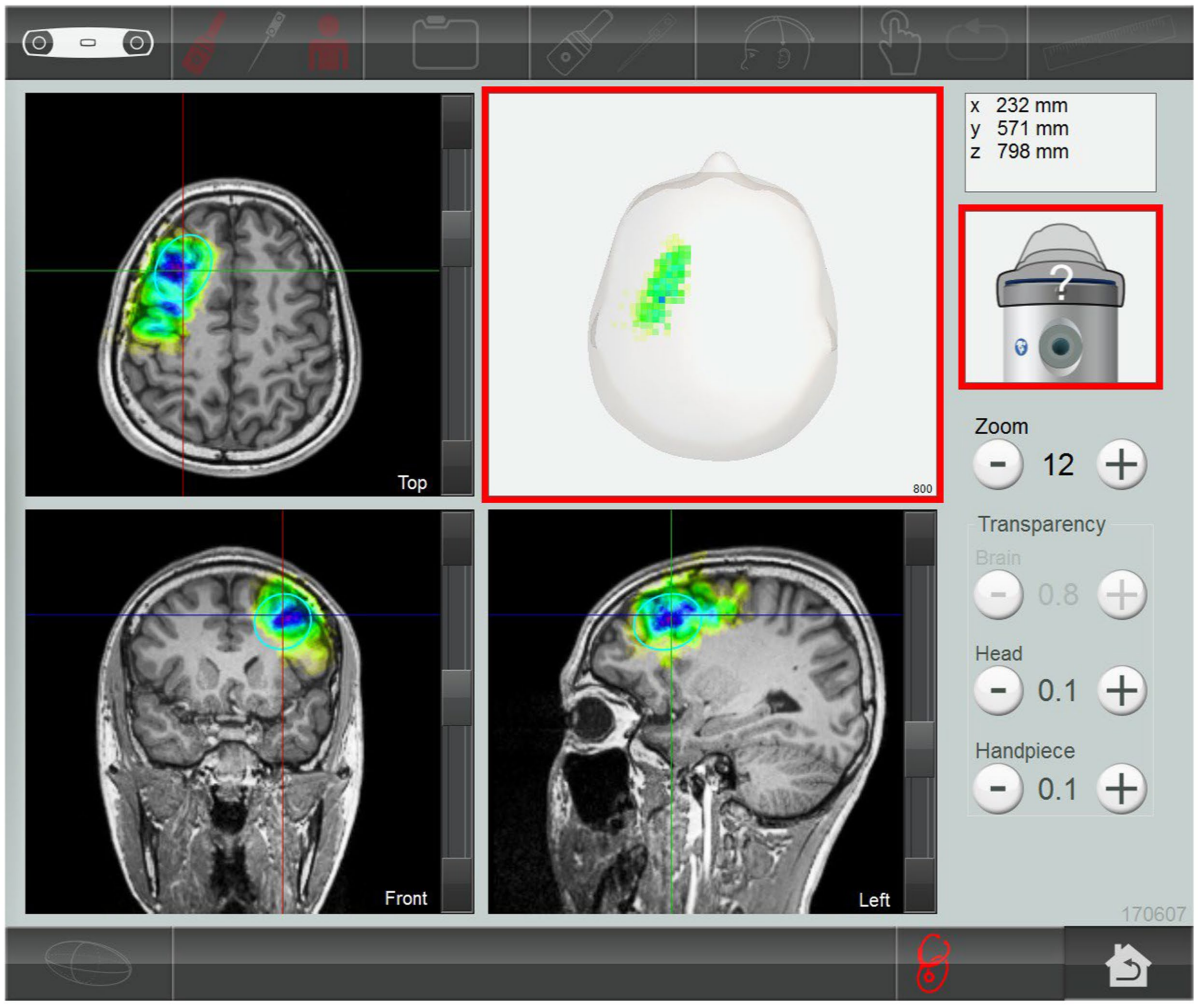
Subject’s MRI T1-weighted images. The stimulated treatment region (left dorsolateral prefrontal cortex) after transcranial pulse stimulation session.

The TPS system consisted of a mobile single transducer and an infrared camera system for MR-based neuronavigation (NEUROLITH, Storz Medical AG, Tägerwilen, Switzerland). During TPS, single ultrashort (3 μs) ultrasound pulses were generated with typical energy levels of 0.2–0.25 mJ/mm2 and pulse frequencies of 4–5 Hz (pulses per second). During the TPS session, participants sat in a comfortable chair in the treatment venue. Participants wore a BodyTrack® system consisting of a 3D camera, tracking glasses with markers, and a TPS handpiece with markers. This BodyTrack® system ensured that the participant’s head matched the T1-weighted images previously obtained at the University Research Facility for Behavioral and Systems Neuroscience (UBSN), PolyU, to allow each TPS pulse to be visualized and documented in real time. Real-time tracking of the handpiece position enabled automatic visualization of the treated brain region. The energy applied is highlighted in green in the figure (Figure 2). The experimenter used the variable stand-offs at the handpiece for depth regulation and manual movement of the handpiece over the skull with real-time visualization on participants’ MRI brain images. The whole treatment session was recorded for *post hoc* evaluation of the locations of the individual intracerebral pulses.

### TPS intervention dose

In this proposed study, we delivered 800 pulses to the subject’s left DLPFC in each session (total: 4,800 pulses). All participants (in both the active and sham TPS groups) received six 30-min TPS sessions over a 2-week period (i.e., 3 sessions/week, on alternate days, total treatment time: 3 h) using energy levels of 0.25 mJ/mm2 and a frequency of 4 Hz. We believe that a two-week TPS intervention is sufficient to test the efficacy of TPS for ADHD (27, 39). Participants were assessed immediately after stimulation (at 2 weeks) and at 1 month and 3 months after the intervention (Figure 1). Also, a posttreatment follow-up at 3 months is sufficient to evaluate the sustainability of TPS for ADHD (27, 39).

For the sham TPS group, participants were given an identical TPS intervention dose, but the silicone oil used in the TPS group was replaced by an air-filled cushion in the handpiece. Participants heard sounds and saw stimuli similar to those of the TPS group.

### Fidelity

To ensure the fidelity of the intervention, the project team ascertained whether the interventions were delivered as intended. The experimenter (PI) has a PhD in Social Sciences (HKU) and is a UK & HK licensed mental health professional with more than 10 years of clinical experience in mental health and neuroscience. The research associates provided WhatsApp message reminders (e.g., of the TPS intervention schedule, fMRI scan appointments, follow-up appointments) to parents to monitor subjects’ progress, adverse effects and treatment adherence throughout the trial period.

### Safety, adverse effects and risk indicators of TPS

TPS uses very low energy for brain stimulation; thus, TPS intervention should not cause any serious adverse effects, such as intracranial bleeding, oedema or other intracranial pathology, as confirmed in previous studies (27, 39). Although this TPS system received clinical certification (CE), indicating that it is a safe intervention, we prepared a checklist of all the potential adverse effects associated with TPS, and monitored subject tolerability and adverse events in each session throughout the trial period. In the pilot RCT on MDD (39), a few subjects reported transient headache (<2 h) (4%), but none required analgesics. Nonetheless, all subjects were covered by master trial insurance in this study.

### Ethical and data security considerations

Participant data from both groups were stored in two separate datasets with an identifier linking these data. Both sets of data were encrypted using TrueCrypt (http://www.truecrypt.org). The data from the baseline and the 12-week follow-up were linked according to personal data. All precautions in data protection were taken, as suggested by TrueCrypt. To prevent leakage of personal data, only the PI had access to the personal dataset. Written consent was obtained from all participants and both of their parents prior to the study. An information sheet containing the purpose of this trial and potential risks and benefits of its procedures regarding MRI scans performed at UBSN/PolyU and TPS was provided to all parents. The parents of participants were informed that their children’s data would be anonymized and that withdrawal or noncompliance would not result in any consequences.

### Measures

#### Demographic data

Basic demographic data, including age, sex, body mass index, years of education, birth history, number of siblings, monthly household income, and first-degree family members’ history of ADHD (yes/no), were collected upon study entry. Details of the subjects’ psychiatric history, including the age at diagnosis and any developmental delays or serious injury of any body parts or serious physical illness (es), were also recorded at the baseline assessment.

#### Attention deficit, hyperactivity impulse, and oppositional defiance

The Swanson, Nolan, and Pelham Teacher and Parent Rating Scale (SNAP-IV) was used to measure inattention, hyperactivity/impulsivity and oppositional defiance. The SNAP-IV consists of 26 items summarized into three factors: inattention, hyperactivity/impulsivity, and oppositional defiance. Based on their general impressions of their children, parents rate the severity of symptoms on a Likert scale (from 0 to 3). A mean score ≤ 1 indicates “normal” or “remission”; a mean score of 1 indicates inattention and hyperactivity/impulsivity; and a mean score ≥ 2 indicates “abnormal.” The SNAP-IV is a reliable and valid scale used in RCT (50) and has good psychometric properties in the Chinese population (51).

#### Clinical global impression (CGI)

The Clinical Global Impression Severity (CGI-S) and Improvement (CGI-I) scales are generally used to assess illness severity and global improvement. The CGI-S is a 7-point clinician-rated scale completed based upon observed and reported symptoms, behavior, and function in the past 7 days. The CGI-I is a 7-point scale used to assess whether the patient’s ADHD has improved or worsened compared to the baseline. These two scales are complementary (52) and have been used in a double-blinded placebo-controlled RCT (53).

#### Executive function

The Stroop test is a neuropsychological test commonly used to assess the inhibitory control component of executive function by testing the subject’s ability to inhibit cognitive interference that occurs when the processing of the target stimulus feature is impeded by the simultaneous processing of a second stimulus attribute (54).

#### ADHD symptoms and behavior

The ADHD Rating Scale–IV (ADHD RS-IV) (55, 56) is a widely used ADHD scale comprising 18 items. This scale is completed by the participant’s parent, who rates the frequency of each symptom. Each item is scored on a 4-point Likert scale (0: never or rarely, 1: sometimes, 2: often, and 3: very often). The 9 odd items evaluate attention deficits, composing the Inattention (IA) subscale; the 9 even items evaluate hyperactivity/impulsivity, composing the Hyperactivity Impulsivity (HI) subscale; the total score is the sum of all the scores on the 18 items. The ADHD-RS-IV is a reliable and valid scale for use in the Chinese population (57).

### Statistical analyses

All statistical analyses were performed using the statistical software R for Windows (R version 4.1.0). Means and standard deviations (SD) of the continuous variables are presented, while numbers and percentages are shown for the categorical variables. A *p* value <0.05 was considered statistically significant. Sociodemographic differences between the TPS group and the sham TPS group were identified using the chi-square test and Student’s *t* test. If there were significant group differences in sociodemographic factors, these variables were considered confounding variables and included as covariates in the analyses. Normality of the primary outcome (SNAP-IV scores) was determined by the Shapiro–Wilk test for each combination of factor levels (group and time). A Student’s t test was used to test the difference in these factors between baseline and the other time points. A linear mixed model was used to examine the group (between-subject factor; TPS and sham TPS), time (within-subject factor), and group × time interaction effects on SNAP-IV scores. *Post hoc* comparisons between groups and time points were conducted using Student’s t tests with Bonferroni correction. The normality of the secondary outcome was determined by the Shapiro–Wilk test at each time point. For normally distributed outcomes, a linear mixed model was used to determine whether the outcome scores significantly differed between pre- and posttest. For outcome scores that grossly deviated from normality, a nonparametric Friedman test was used to determine the mean difference. The effect size of each outcome (Cohen’s *d*) was calculated, with *d* = 0.2, 0.5, and 0.8 corresponding to small, medium, and large effect sizes (58). Missing data were managed by multiple imputation (59).

## Results

### Sociodemographic differences between the TPS and sham TPS groups

There were no statistically significant differences in sociodemographic characteristics between the TPS group and the sham TPS group (all *p* > 0.05). The mean age of the participants was 13.1 years (SD = 1.44). There were more male participants (78%) than female participants. All participants were currently taking medication (methylphenidate HCL), with more than half of the participants (56%) reporting good drug compliance and 62% reporting adverse effects after taking medication. Of these participants, 43% (n = 5) had a family history of psychiatric disorders (ADHD, dyslexia, MDD, anxiety disorder, Asperger’s disease), 34% (n = 11) had siblings with psychiatric disorders/problems (i.e., autism spectrum disorder, ADHD, dyslexia, and language delay), 81% had married parents, 94% had obtained secondary education or above, and 59% had a parent that was a homemaker. Other participants (41%) had parents working in semiskilled occupational sectors (see Supplementary Table S1).

### Adverse effects, safety issues, and treatment compliance

Overall, three subjects in the TPS group reported transient mild headache during TPS administration, with a mean pain score of 4 out of 10 (range: 0 = no pain to 10 = very severe pain). The pain duration was less than 3 min. No analgesics were required by any subjects, and no parents reported any adverse effects after TPS to the research team. No subjects/parents reported adverse effects in the sham TPS group. In this study, the attrition rate was 0% at all time points. The treatment compliance rate was 100%, which is considered highly encouraging.

### Effects of TPS

None of the primary and secondary outcome scores were normally distributed, as shown in Table 2.

**Table 2:** Normality of the primary and secondary outcome scores tested by the Shapiro–Wilk test for each time point.

	Overall	Baseline	Posttest	1-month f/u	3-month f/u
	*p*	*p*	*p*	*p*	*p*
SNAP-IV total SNAP-IV mean score ADHD RS-IV Strooptest1 (reaction time) Strooptest2 (reaction time) Strooptest3 (reaction time) DS-Forward DS-Backward DS-Forward (length) DS-Backward (length) CGI-Severity CGI-Improvement CGI-Efficacy CGI-Total	0.009** 0.02* 0.02* <0.001*** <0.001*** <0.001*** <0.001*** <0.001*** <0.001*** <0.001*** <0.001*** <0.001*** <0.001*** <0.001***	0.75 <0.001*** 0.38 <0.001*** 0.05* <0.001*** 0.004** 0.04* <0.001*** <0.001*** <0.001*** <0.001*** <0.001*** <0.001***	0.06 0.33 0.39 0.10 0.22 0.006** <0.001*** 0.12 <0.001*** 0.006** <0.001*** <0.001*** <0.001*** <0.001***	0.51 0.22 0.89 <0.001*** 0.03* <0.001*** 0.002** 0.11 0.89 0.008** <0.001*** <0.001*** <0.001*** 0.05	0.41 0.35 0.55 0.03* <0.001*** 0.02* <0.001*** 0.27 0.24 0.56 <0.001*** <0.001*** <0.001*** <0.001***

SNAP-IV, Swanson, Nolan, and Pelham Teacher and Parent Rating Scale; ADHD RS-IV, ADHD Rating Scale–IV; DS, digit span; CGI, clinical global impression.

***p < 0.001;

**p < 0.01;

*p < 0.05.

Table 3 shows the group, time, and group × time interaction effects on the primary (SNAP-IV) and secondary outcomes (scores on the ADHD-RS-IV, Stroop test, digit span test (forwards and backwards) and the CGI-S, CGI-I, and CGI total) in the TPS group and the sham TPS group. There were significant interaction effects on scores on the SNAP-IV, ADHD-RS-IV, DS forward (length), CGI-S, CGI-I, and CGI total as well as on reaction times on the Stroop test in the word reading (test 1), colour naming (test 2), and named colour-word (test 3) conditions (all *p* < 0.05). There was no group difference in primary or secondary outcome scores at baseline (*p* > 0.05).

**Table 3:** The group, time, and group x time interaction effects of the outcomes between the TPS group and the sham TPS group.

	Group	Time	Group x time
	*p*	*p*	*p*
SNAP-IV mean score ADHD RS-IV Strooptest1 (reaction time) Strooptest2 (reaction time) Strooptest3 (reaction time) DS-Forward DS-Backward DS-Forward (length) DS-Backward (length) CGI-Severity CGI-Improvement CGI-Efficacy CGI-Total	0.94 0.26 0.22 0.11 0.04* 0.60 0.003** 0.71 0.03* 0.82 0.63 0.41 0.33	<0.001*** <0.001*** 0.61 0.04* 0.18 0.89 0.75 0.29 0.49 0.10 <0.001*** 0.16 <0.001***	<0.001*** <0.001*** 0.03* 0.02* <0.001*** 0.71 0.07 <0.001*** 0.06 0.002** <0.001*** 0.69 <0.001***

Adjusted for age, gender, and drug compliance. SNAP-IV, Swanson, Nolan, and Pelham Teacher and Parent Rating Scale; ADHD RS-IV, ADHD Rating Scale–IV; DS, digit span; CGI, clinical global impression.

***p < 0.001;

**p < 0.01;

*p < 0.05.

**Figure 3:**
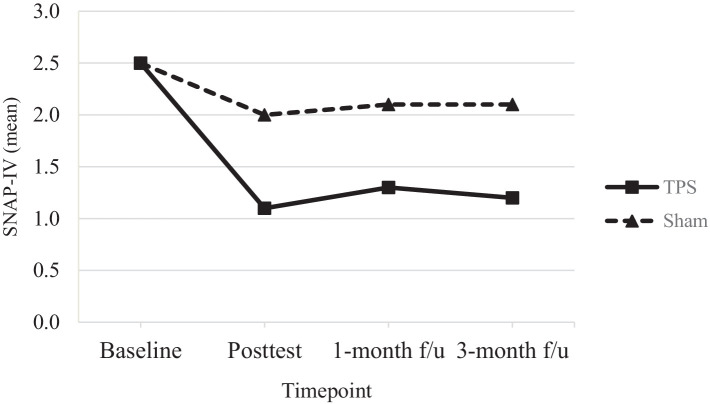
Mean scores of SNAP-IV of ADHD participants in TPS and Sham group at different study time points.

**Figure 4:**
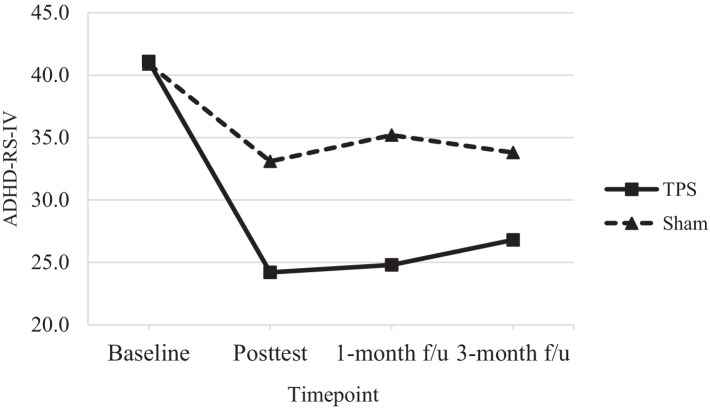
Total scores of ADHD-RS-IV of ADHD participants in TPS and Sham group at different study time points.

**Figure 5:**
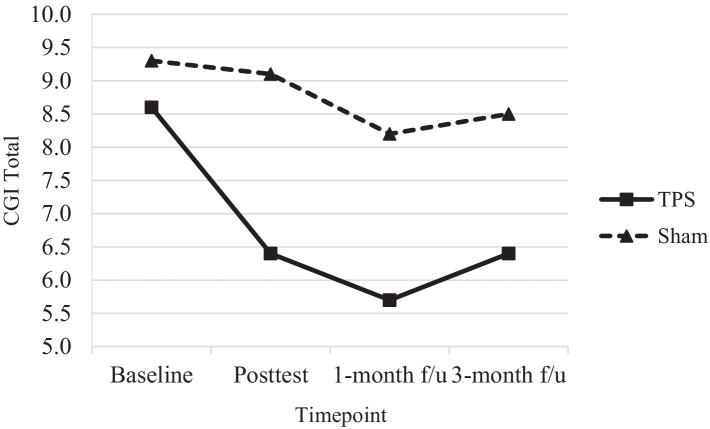
CGI total scores of ADHD participants in TPS and Sham group at different study time points.

Table 4 shows the results of *post hoc* comparisons between groups at each time point to further elucidate the interaction effects on SNAP-IV (see also Figure 3), ADHD-RS-IV (see also Figure 4), CGI-I, and CGI total scores (see also Figure 5). The TPS group had significantly lower mean SNAP-IV scores at posttest (T2), with a large effect size (*d* = 0.75) (*d* = 2.45). Additionally, the TPS group also had significantly lower SNAP-IV scores at the 1-month and 3-month follow-ups (all *p* < 0.001) than the sham TPS group. The effect of group on the primary outcome (SNAP-IV scores) was medium to large (Cohen’s *d* values at posttest, 1-month follow-up, and 3-month follow-up: 2.32, 2.45, and 2.40, respectively). Regarding secondary outcomes, the effect on ADHD-RS-IV (*d* = 1.04), CGI-I (*d* = 1.04–5.63), and CGI total scores was large (*d* = 1.13–2.69).

**Table 4:** *Post-hoc* comparisons between the TPS group and the sham TPS group at each time point.

	Time	TPS Mean (SD)	Sham TPS Mean (SD)	*p*
SNAP-IV (mean)	Baseline	2.5 (0.20)	2.5 (0.24)	0.83
	Posttest	1.1 (0.34)	2.0 (0.43)	0.00***
	1-month f/u	1.3 (0.30)	2.1 (0.35)	0.00***
	3-month f/u	1.2 (0.39)	2.1 (0.36)	0.00***
ADHD RS-IV	Baseline	41.1 (7.27)	40.9 (8.13)	1.00
	Posttest	24.2 (12.28)	33.1 (12.41)	0.07
	1-month f/u	24.8 (10.85)	35.2 (9.08)	0.01*
	3-month f/u	26.8 (13.21)	33.8 (10.34)	0.14
Strooptest1 (reaction time)	Baseline	18.5 (9.58)	14.6 (3.76)	0.05
	Posttest	14.4 (3.62)	15.2 (4.60)	0.79
	1-month f/u	14.1 (5.60)	13.0 (3.34)	0.77
	3-month f/u	12.7 (3.72)	13.2 (3.77)	0.74
Strooptest2 (reaction time)	Baseline	19.2 (5.05)	17.0 (4.99)	0.15
	Posttest	17.1 (4.32)	14.8 (4.31)	0.12
	1-month f/u	15.6 (4.01)	14.0 (4.81)	0.13
	3-month f/u	18.2 (11.13)	14.0 (4.88)	0.18
Strooptest3 (reaction time)	Baseline	29.5 (10.32)	28.0 (15.48)	0.35
	Posttest	26.7 (11.82)	22.0 (8.41)	0.26
	1-month f/u	24.5 (12.46)	18.2 (5.41)	0.11
	3-month f/u	20.0 (7.67)	17.8 (7.10)	0.33
DS-Forward	Baseline	12.3 (1.21)	12.2 (1.70)	0.94
	Posttest	12.2 (1.92)	12.7 (1.23)	0.75
	1-month f/u	12.6 (1.42)	12.5 (1.25)	0.76
	3-month f/u	12.5 (2.04)	12.7 (1.16)	0.81
DS-Backward	Baseline	6.4 (2.96)	7.1 (3.79)	0.76
	Posttest	7.8 (3.03)	7.6 (3.78)	0.73
	1-month f/u	7.4 (2.60)	8.7 (3.50)	0.25
	3-month f/u	8.9 (2.83)	8.5 (3.50)	0.84
DS-Forward (length)	Baseline	5467.1 (558.39)	5332.0 (1367.06)	0.33
	Posttest	4867.1 (711.79)	5008.0 (1531.36)	0.66
	1-month f/u	4482.4 (785.68)	4852.0 (839.35)	0.27
	3-month f/u	4065.9 (865.99)	4244.0 (521.82)	0.50
DS-Backward (length)	Baseline	5371.8 (3245.56)	5420.0 (4798.00)	0.46
	Posttest	6878.8 (4065.36)	6840.0 (4722.72)	0.78
	1-month f/u	6102.4 (2983.96)	7468.0 (3950.89)	0.33
	3-month f/u	8209.4 (3202.09)	7080.0 (3153.69)	0.44
CGI-Severity	Baseline	4.5 (0.62)	4.5 (1.19)	0.60
	Posttest	4.1 (0.90)	4.3 (0.70)	0.72
	1-month f/u	3.7 (0.59)	4.1 (0.74)	0.10
	3-month f/u	4.4 (0.49)	4.4 (0.51)	0.80
CGI-Improvement	Baseline	4.0 (0.00)	4.0 (0.00)	>0.99
	Posttest	2.3 (0.77)	4.0 (0.00)	0.00***
	1-month f/u	1.9 (0.43)	3.9 (0.26)	0.00***
	3-month f/u	1.9 (1.09)	4.0 (0.00)	0.00***
CGI-Efficacy	Baseline	0.1 (0.24)	0.9 (3.36)	0.93
	Posttest	0.0 (0.00)	0.8 (2.83)	0.14
	1-month f/u	0.1 (0.24)	0.1 (0.35)	0.50
	3-month f/u	0.1 (0.33)	0.1 (0.26)	0.65
CGI-Total	Baseline	8.6 (0.62)	9.3 (3.94)	0.52
	Posttest	6.4 (1.37)	9.1 (3.10)	0.00***
	1-month f/u	5.7 (0.92)	8.2 (0.94)	0.00***
	3-month f/u	6.4 (1.23)	8.5 (0.52)	0.00***

SNAP-IV, Swanson, Nolan, and Pelham Teacher and Parent Rating Scale; ADHD RS-IV, ADHD Rating Scale–IV; DS, digit Span; CGI, clinical global impression.

***p < 0.001;

*p < 0.05.

### Blinding

In this study, parents were asked to guess the group in which their children were placed to determine the success of the blinding procedures, as some subjects had some difficulty in understanding the concept of blinding. In the TPS group, 76.5% (*n* = 13 out of 17) guessed correctly, while in the sham TPS group, 46.7% (*n* = 7 out of 15) guessed correctly, indicating that our blinding process was successful.

Since some parents in the sham TPS group believed that their children had received the TPS, we analyzed the blinding success between the two groups using the *x*2 test (3.20); the result was not significant (*p* = 0.08), indicating that parents’ belief that their child had received active stimulation was not dependent on actual group allocation, confirming that the effect of TPS was solely due to the actual stimulation rather than a placebo effect.

## Discussion

This study is the first RCT to evaluate the efficacy and safety of TPS for ADHD in Chinese young adolescents. Notably, we found that TPS improved ADHD core symptoms, and the effects were sustained at the 1- and 3-month follow-ups. Our results are supported by a recent double-blind, sham-controlled trial administering transcranial random noise stimulation (tRNS) (60) to 23 children aged 6 to 12 years. Subjects received 10 sessions of tRNS over the inferior frontal gyrus (rIFG) and left dorsolateral prefrontal cortex (lDLPFC) plus cognitive training (CT) over 2 weeks. The authors reported that tRNS was effective in reducing ADHD symptoms (evaluated by ADHD-RS-IV scores), as revealed by a comparison of the tRNS+CT group and the sham tRNS+CT group. The posttreatment effect size was *d* = 2.4 and dropped to 1.7 at the 3-week follow-up.

Our findings may substantially impact patients and their caregivers as well as the larger community. These results can inform health policymakers regarding the ability to use TPS as an adjunct treatment in the clinical setting in psychiatry, given that both conventional treatments (medication and psychotherapy) involve long-term input to sustain the therapeutic effects in individuals with ADHD. These treatment methods inevitably increase health costs, the caregiving burden and the global disease burden. We showed that TPS is effective in the treatment of ADHD patients, providing hope for patients’ families and reducing their psychological burden to a large extent because ADHD is curable and treatable by TPS. This represents a breakthrough in neuroscience research for adolescents with special education needs (SEN) in Hong Kong.

### Primary outcome measure

#### Snap-IV

In the TPS group, mean SNAP-IV scores (a measure of ADHD symptom severity) exhibited a 44% reduction by the posttreatment time point; in the sham TPS group, a 20% reduction in these scores was observed. There was a further reduction in the scores of the TPS group at the 1-month (52%) and 3-month (48%) follow-ups, while the sham TPS group exhibited a 16% reduction at both of these time points. The changes in SNAP-IV scores significantly differed between the TPS and sham TPS groups at posttreatment and at the 1-month and 3-month follow-ups (all *p*s < 0.001). In addition, the effect size was large, with Cohen’s *d* values ranging from 2.32 (posttreatment) to 2.45 (1-month follow-up) and 2.40 (3-month follow-up) (Table 4).

### Secondary outcome measures

#### ADHD-RS-IV

A 30% reduction in ADHD-RS-IV scores is considered to reflect a clinically acceptable ADHD treatment response (61, 62). We found that 41.1% of participants in the TPS group achieved a clinically effective treatment response at posttreatment compared to 19.1% of participants in the sham TPS group. In addition, 39.7 and 35% of participants in the TPS group achieved an effective treatment response at the 1-month and 3-month follow-ups, respectively, while 13.9 and 17.4% of participants in the sham TPS group achieved such a response (Table 4). The changes in ADHD-RS-IV scores were marginally significant at posttreatment (*p* = 0.07), significant at the 1-month follow-up (*p* = 0.01), and nonsignificant at the 3-month follow-up (*p* = 0.14).

The study showed an initial and 1-month post-treatment improvement in fundamental ADHD symptoms, but this was not maintained at the 3-month follow-up. The likely reason for this is that the initial TPS protocol was only an estimate, as no previous TPS studies had targeted the ADHD population. Factors such as the total number of stimulation pulses and the pulse repetition rate could influence the effectiveness of the TPS treatment for ADHD symptoms. The findings suggested that these TPS parameters are safe for use in young adolescents, with effects gradually appearing after stimulation. However, the energy supplied may not be enough to modify all ADHD symptoms. This underscores the need for larger-scale research and the development of a standard protocol to maximize therapeutic benefits for the ADHD population.

#### Stroop test (1, 2, 3), digit span test (forwards/backwards), digit span (length), CGI-S, CGI-E

There were no statistically significant effects of group on scores on the Stroop test (1, 2, 3), digit span (forwards/backwards), digit span (length), CGI-S, or CGI-E (all *P*s > 0.05).

#### CGI-I and CGI total scores

Nonetheless, both the mean CGI-I and mean CGI total scores significantly differed between the TPS group and the sham TPS group at posttreatment and at the 1-month and 3-month follow-ups (all *P*s < 0.001). The CGI-I and CGI total scores were provided by the interventionist, and it is encouraging to note that parental ratings of improvement in the TPS group were in line with the professional assessment of the experimenter at all time points.

Our findings regarding the primary outcome (SNAP-IV scores) and one of the secondary outcomes (ADHD-RS-IV scores) of this study are highly encouraging, as both were parent-reported scales that yielded statistically significant differences in the TPS group at all time points. However, there was no effect of treatment on the other secondary measure (that reported by the subjects), particularly in terms of working memory and executive function (EF). This null effect may be explained by the fact that changes in EF may require pharmaceutical input and psychotherapy (for parents/children) over a period of 1 to 3 months (63–66). In other words, monotherapy or TPS alone may have less effect on EF within a short period. The lack of significant difference between the TPS and Sham groups, leading to inconclusive results, could be due to the possible placebo effect impacting the sham participants. This is especially relevant in the context of a randomized-controlled study design (67). In addition, drug adherence may also contribute to the efficacy of TPS in our subjects. Presumably, all subjects took their prescribed medications regularly, but on some occasions, some subjects may have struggled to comply with their current medication regimen due to COVID-19 symptoms (e.g., fever, coughing, physical exhaustion) during the intervention period, despite parental/medical advice. We also speculate that the null effect may also be attributed to the relatively mild or moderate symptom severity and mild executive dysfunction in this sample, as all our subjects were enrolled in mainstream schools in Hong Kong; hence, the effect of treatment on EF may be less prominent in our study. Our results are in line with a pilot study (68) which evaluated the effect of tDCS and tRNS on ADHD symptoms. However, there is no consensus regarding the optimal treatment region in the brain for the treatment/management of ADHD symptoms (49); previous neuroscientific research seems to target the bilateral DLPFC (69), lDLPFC (4), rIFC (70), IFC-parieto-cerebellar networks or prefrontal striatal circuits (71). Future studies should determine the optimal TPS protocol and parameters to yield EF changes in the ADHD population.

In our ADHD protocol (72), we have mentioned that most NIBS studies on ADHD have used EEG-neurofeedback and rTMS/tDCS across different age groups but have yielded inconsistent results in this population. More importantly, most all NIBS studies primarily focused on left/right/bilateral DLPFC (dorsal lateral prefrontal cortex) in ADHD, possibly due to the fact that brain stimulation targeting the right inferior frontal cortex (rIFC) was shown to be ineffective (23). Some studies showed that brain stimulation over the left DLPFC improved the response inhibition, attention, working memory, and cognitive flexibility in ADHD patients (73). Since patients with attention deficit hyperactivity disorder (ADHD) are characterized by both underactivation of the prefrontal cortex and deficits in Working Memory (WM), the modulation of prefrontal activity with TPS in ADHD patients may increase their WM performance as well as improve the activation and connectivity of the WM network (73). Thus, our study findings suggested that TPS caused increased neuronal activation and connectivity, not only in the targeted brain treatment region (i.e., left DLPFC) but also in other remote brain regions which will be covered in another paper which used neuroimaging to evaluate the efficacy of TPS with resting-state MRI (Cheung et al. (17), under review).

At present, there is no standardized TPS protocol on various neurodegenerative diseases and neurodevelopmental disorders. We have reviewed existing TPS randomized controlled trials and other open-label studies with the conclusion (see Supplementary Table S2) that, different non-invasive brain stimulation techniques using RCT sham-controlled design on different ages and clientele seems to demonstrate inconsistent findings. Using our previous double-blind randomized, sham-controlled RCT on ASD as an example, participants in the verum TPS group had a significant change in the CARs score (primary outcome) immediately after 2-week TPS, and at 1- month and 3-month follow-up compared to the sham TPS. Nonetheless, in this study, participants had significant improvement in the SNAP-IV score, but there was no significant improvement in the ADHD RS-IV in the verum TPS group immediately after the 2-week intervention but became significant again at the post-stimulation at 1-month follow-up and not significant again at 3-month follow-up, when compared to the sham-controlled group. Such intriguing results may be generated from the following speculations:

1. survey/respondent fatigue, which is a well-documented phenomenon (74) when participants are tired of the survey task and the quality of the data may be affected. In this study, ADHD RS-IV was the second psychological instrument which sequentially followed by SNAP-IV. These two sets of surveys have some questions in common and it is plausible that participants’ parents were tired when they were asked to answer similar questions in written form which could bias their subjective results toward the participants.
2. Lack of significance between TPS group and the sham TPS group may also attributed to placebo effects in the latter group. Participants in the sham TPS group may have the belief and desire that they were administered the TPS during the treatment process and such belief/desire may bias their subjective data in the self-reported survey (67).

### Limitations of the study

Although our study findings demonstrated that TPS is an effective NIBS in the treatment of some ADHD symptoms, there are some limitations that should be addressed. First, this study was a single-site study in Hong Kong with a relatively small sample size. Thus, the findings may not be translatable or generalizable to other country/cultural contexts. Second, we included only subjects enrolled in mainstream schools, and it is not known whether TPS also benefits ADHD patients with severe/very severe symptoms who attend special schools. Third, future studies should include cognitive training in the intervention and use a larger sample size to ascertain whether TPS can be a standalone adjunct treatment. Fourth, despite all participants declared taking prescribed medications throughout the intervention period, only 56% reported good medication adherence and thus, the mean dosage of the medication was not considered as a reliable variable in the statistical analysis.

## Conclusion

Our findings provide new understanding and insight into the field of neuroscience. We demonstrated that TPS is an effective, safe, and scientific NIBS that can be used to treat most (but not all) ADHD core symptoms. The long-term effects of TPS require further investigation in multi-national trials. Nevertheless, the incorporation of TPS as a potential means of adjunct treatment option for ADHD should be considered by health policymakers in the near future.

## Data availability statement

The original contributions presented in the study are included in the article/Supplementary material, further inquiries can be directed to the corresponding author.

## Ethics statement

The studies involving humans were approved by Institutional Review Board, The Hong Kong Polytechnic University. The studies were conducted in accordance with the local legislation and institutional requirements. Written informed consent for participation in this study was provided by the participants’ legal guardians/next of kin.

## Author contributions

TC: Conceptualization, Funding acquisition, Investigation, Methodology, Resources, Supervision, Writing – original draft, Writing – review & editing. BY: Formal analysis, Investigation, Methodology, Validation, Writing – original draft, Writing – review & editing. BC: Investigation, Validation, Writing – review & editing. JL: Data curation, Formal analysis, Investigation, Project administration, Writing – original draft, Writing – review & editing. KF: Data curation, Formal analysis, Investigation, Project administration, Writing – original draft, Writing – review & editing. HL: Investigation, Writing – review & editing. TL: Formal analysis, Investigation, Methodology, Writing – review & editing. AL: Investigation, Writing – review & editing. LS: Investigation, Writing – review & editing. RB: Conceptualization, Investigation, Validation, Writing – review & editing. CC: Conceptualization, Investigation, Methodology, Validation, Writing – review & editing.
