# Supplementary material for: Efficacy and safety of transcranial pulse stimulation in young adolescents with attention-deficit/hyperactivity disorder: a pilot, randomized, double-blind, sham-controlled trial
Source: Front Neurol. 2024 May 9;15:1364270. doi: 10.3389/fneur.2024.1364270 (PMC11112118; doi:10.3389/fneur.2024.1364270)
Supplement: Supplementary file 1 [file Table_1.DOCX]

**Supplemental Material**

**Supplementary Table S1.** Differences in sociodemographic characteristics between the TPS group and the sham TPS group.

| Participant sociodemographic characteristic | Intervention (n=17) | Sham (n=15) | *p* |
| --- | --- | --- | --- |
| Age  Sex  Male  Female  Education level  Senior primary  Junior high school  Senior high school  Academic performance  Good  Fair  Poor  Mean duration of ADHD diagnosis (years)  Current medication use  Yes  Mean age of starting medication  Duration of medication use (months)  Drug compliance  Good  Fair  Poor  Adverse effects of medication  Yes  No  Number of siblings  Family history of psychiatric disorders*  Yes  No | 12.8 (1.51)  13 (76.47)  4 (23.53)  3(17.6)  13 (76.5)  1 (5.9)  1 (5.88)  8 (47.06)  8 (47.06)  6.71 (2.89)  17 (100)  8 (2.09)  64.24 (26.13)  9 (52.94)  5 (29.41)  3 (17.65)  9 (52.94)  8 (47.06)  1.4 (1.00)  7 (41.18)  10 (58.82) | 13.3 (1.34)  12 (80.00)  3 (20.00)  2 (13.3)  12 (80)  1 (6.7)  1 (6.67)  8 (53.33)  6 (40.00)  6.80 (2.46)  15 (100)  7.13 (1.85)  73.07 (36.14)  9 (60.00)  3 (20.00)  3 (20.00)  11 (73.33)  4 (26.67)  1.3 (1.11)  6 (40.00)  9 (60.00) | 0.21  1.00  0.94  0.92  0.80  1.00  0.16  0.39  0.83  0.41  0.82  1.00 |
| Parental sociodemographic characteristics  Marital status  Married  Separated/divorced/widowed  Single  Education  Primary education or below  Secondary education  Associate degree  Bachelor’s degree or above  Occupation  Finance sector  Catering  Government  Homemaker/student  Insurance agent/sales/property management  IT/technician  Registered nurse (General) | 13 (76.47)  3 (17.65)  1 (5.88)  2 (11.76)  6 (35.29)  5 (29.41)  4 (23.53)  0 (0.00)  1 (5.88)  0 (0.00)  8 (47.06)  3 (17.64)  3 (17.64)  1 (5.88) | 12 (80.00)  3 (20.00)  0 (0.00)  0 (0.00)  8 (53.33)  5 (33.33)  2 (13.33)  2 (13.34)  0 (0.00)  1 (6.67)  11 (73.33)  1 (6.67)  0 (0.00)  0 (0.00) | 0.55  0.42  0.41 |

*Note.* *First-degree relative, including parents and siblings

**Supplementary Table S2.** Current Findings of Transcranial Pulse Stimulation (TPS) studies

| **Author (Year);**  **Place** | **Study Design** | **Subjects** | **N** | **Age** | **TPS Parameters** | **Duration** | **Stimulation Region** | **Outcome Measures** | **Major Findings** |
| --- | --- | --- | --- | --- | --- | --- | --- | --- | --- |
| Beisteiner et al., (2020); Austria | Open-label | AD | 35  (15 males) | 51-84 | 6000 pulses per session (3 μs),  energy flux density = 0.2 mJ/mm^2^  pulse repetition rate = 5 Hz pulse/sec | 2 – 4 weeks,  3 sessions per week  (Total: 6 sessions) | Bilateral frontal cortex and lateral  parietal cortex, and extended  precuneus cortex | CERAD total score,  CERAD logistic  regression score,  CERAD principal  component  analysis, fMRI,  GDS, BDI-II | - Large safety margins and dose-dependent neuromodulation.  - High treatment tolerability and no major side effects.  - Significant improvement in neuropsychological scores and improvement lasts three months.  - Neuropsychological scores correlates with an upregulation of the memory network. |
| Popescu et al. (2021); Austria | Open-label | AD | 17 | Nil |  |  |  | CERAD total score, fMRI | - Significant correlation between neuropsychological improvement and increased cortical thickness in AD-critical brain areas. |
| Dörl et al. (2022); Austria | Open-label | AD | 18  (7 males) | x̄ =  69.9 |  |  |  | CERAD figural  score, fMRI | - Significant improvement in 3-month follow-up CERAD score.  -Increased functional connectivity in hippocampal level. |
| Matt et al. (2022); Austria | Open-label | AD | 18 | Nil |  |  |  | BDI-II, fMRI | - Decrease in depressive symptoms and improvement in BDI scores. |
| Cont et al. (2022); Germany | Open-label | AD | 11  (9 males) | 59-77 | 6000/3000 pulses per session (3 μs),  energy flux density = 0.2 mJ/mm^2^  pulse repetition rate = 4 Hz pulse/sec | 2 weeks (6 sessions, 6000 pulses) or  2 weeks (12 sessions, 3000 pulses) | Bilateral frontal, lateral parietal, and temporal cortex, extended precuneus  cortex | ADAS score, MMSE, MoCA, NRS | - TPS is safe and well tolerated.  - Global cognitive improved in total ADAS score. |
| Sprick & Köhne (2022); Germany | Open-label | AD | 21 | 59-86 | 6000 pulses per session (3 μs),  energy flux density = 0.25 mJ/mm^2^  pulse repetition rate = 4 Hz pulse/sec | 2 weeks,  3 sessions per week  (Total: 6 sessions) | Left and right dorsolateral frontal cortex, parietal and temporal lobes | Stroop tests,  BDI-II | - Improvement in executive functioning and alleviated depressive symptoms (significant improvement in Stroop tests results and decrease in BDI score) |
| Fong et al. (2023); Hong Kong | Open-label | NCD | 19  (7 males) | x̄ =  74.3 | 6000 pulses per session (3 μs),  energy flux density = 0.2-0.25 mJ/mm^2^  pulse repetition rate = 4-5 Hz pulse/sec | 2 weeks,  3 sessions per week  (Total: 6 sessions) | Frontal, parietal,  temporal, and occipital lobes | HK-MoCA, VFT, Stroop, TMT, DS, HDRS-17, AES-C, Chinese IADL, APOE genotype, BDNF | - Improvement in cognitive functioning (significant improvement in HK-MoCA after TPS intervention) |
| Osou et al. (2023); Austria | Open-label | PD | 20  (15 males) | 48-84 | 4000 pulses per session (3 μs)  energy flux density = 0.25 mJ/mm^2^  pulse repetition rate = 5 Hz pulse/sec | 2 weeks,  5 sessions per week  (Total: 10 sessions) | Primary sensorimotor, supplementary  motor, cingulate motor area | UPDRS‑III | - Improvement in motor symptoms (significant improvement in UPDRS-III scores) |
| Cheung, Li, Ho, et al., (2023); Hong Kong | Single-blinded RCT | MDD | 30  (8 males) | 18-54 | 300 pulses per session (3 μs)  energy flux density = 0.2-0.25 mJ/mm^2^  pulse repetition rate = 3-4 Hz pulse/sec | 2 weeks,  3 sessions per week  (Total: 6 sessions) | Left dorsal lateral prefrontal cortex  (L-DLPFC) | HDRS-17, SHAPS, Chinese IADL, HK-MoCA, DS, TMT | - Significant reduction in the depressive symptom severity in the TPS group as compared to the waitlist-controlled group |
| Cheung, Li, Lam, et al., (2023); Hong Kong | Double-blinded RCT | ASD | 32  (27 males) | 12-17 | 800 pulses per session (3 μs)  energy flux density = 0.2-0.25 mJ/mm^2^  pulse repetition rate = 2-4 Hz pulse/sec | 2 weeks,  3 sessions per week  (Total: 6 sessions) | Right temporoparietal junction (rTPJ) | CARS, AQ, SRS, ASAS, TMT, VFT, Stroop, DS, CGI | - Reduction the core symptoms of autism spectrum disorder  - some neurocognitive improvement in 1-month follow-ups |
| Cheung, Yee, et al., (2023); Hong Kong | Double-blinded RCT | ADHD | 32  (25 males) | 12-17 | 800 pulses per session (3 μs)  energy flux density = 0.2-0.25 mJ/mm^2^  pulse repetition rate = 2-4 Hz pulse/sec | 2 weeks,  3 sessions per week  (Total: 6 sessions) | Left dorsal lateral prefrontal cortex  (L-DLPFC) | SNAP-IV, ADHD-RS-IV, Stroop, DS, CGI | - 30% reduction of ADHD symptoms  - effect sustained through the 1- and 3-month follow-up periods |

Abbreviations: RCT, Randomized Controlled Trials; AD, Alzheimer’s Disease; NCD, Mild Neurocognitive Disorders; PD, Parkinson’s Disease; MDD, Major Depressive Disorder; ASD, Autism Spectrum Disorder; ADHD, Attention-Deficit/Hyperactivity Disorder; CERAD, Consortium to Establish a Registry for Alzheimer's Disease; fMRI, functional Magnetic Resonance Imaging; ADAS, Alzheimer's Disease Assessment Scale; GDS, Geriatric Depression Score; MMSE, Mini-Mental Status Examination; MoCA, Montreal Cognitive Assessment; NRS, Numeric Rating Scale; BDI, Beck Depression Inventory; VFT, Verbal Fluency Test; TMT, Trail Making Test; DS, Digit Span; HDRS, Hamilton Depression Rating Scale; AES-C, Apathy Evaluation Scale-Clinician; IADL, Instrumental Activities of Daily Living; BDNF, Brain-derived Neurotrophic Factor; UPDRS-III, Unified Parkinson's Disease Rating Scale-Part 3; SHAPS, Snaith-Hamilton Pleasure Scale; CARS, Childhood Autism Rating Scale; AQ, Autism Spectrum Quotient; SRS, Social Responsiveness Scale; ASAS, Australian Scale for Asperger’s Syndrome; CGI, Clinical Global Impression; SNAP-IV, Swanson, Nolan, and Pelham Teacher and Parent Rating Scale; ADHD-RS-IV, ADHD Rating Scale–IV.
